# Cannabidiol plasma determination and pharmacokinetics conducted at beginning, middle and end of long-term supplementation of a broad-spectrum hemp oil to healthy adult dogs

**DOI:** 10.3389/fvets.2023.1279926

**Published:** 2023-09-29

**Authors:** Isabella Corsato Alvarenga, Daniel Gustafson, Krista Banks, Kim Wilson, Stephanie McGrath

**Affiliations:** ^1^Department of Clinical Sciences, College of Veterinary Medicine and Biomedical Sciences, Colorado State University, Fort Collins, CO, United States; ^2^Hill’s Pet Nutrition, Topeka, KS, United States

**Keywords:** CBD, hemp, PK, canine, long-term, health

## Abstract

**Introduction:**

Veterinary hemp products containing cannabidiol (CBD) and negligible psychoactive (THC) have increased popularity since hemp (with <0.3% THC) was removed from schedule 1 substances under the Controlled Substances Act in 2018. This was accompanied by increased CBD research, mostly on the short-term safety and efficacy for inflammatory and neurological conditions. It is imperative to understand how CBD is metabolized or accumulated in the body long-term, thus the goal of the present work was to determine monthly plasma CBD concentrations, as well as changes in pharmacokinetic (PK) parameters in chronically dosed dogs.

**Methods:**

The study was a masked, placebo-controlled, randomized design. Six adult beagles were assigned to placebo, 5 and 10 mg/kg/day CBD treatment groups. Dogs received oral oil treatment once daily for 36 weeks. Blood was collected once every 4 weeks pre- and postprandially for CBD plasma determination (at 0 and 2 h). Pharmacokinetics were conducted at 0, 18 and 36 weeks. Pharmacokinetics and monthly CBD plasma data of dogs who received CBD were analyzed as repeated measures over time using a mixed model, with significance at *α* = 0.05.

**Results:**

Average plasma CBD at 5 and 10 mg/kg were 97.3 ng/mL and 236.8 ng/mL pre-prandial, 341 ng/mL and 1,068 ng/mL postprandial, respectively. PK parameters suggested CBD accumulation over time, with significant increases in C_max_ and AUC at both the 18 and 36-week timepoints. C_max_ and AUC were dose proportional. Half-life demonstrated large inter-individual variations and increased (*p* < 0.05) at weeks 18 and 36 compared to baseline. Volume of distribution was not affected by time or treatment, while MRT increased, and clearance decreased over time (*p* < 0.05).

**Conclusions and clinical importance:**

Chronic administration of CBD to healthy adult dogs led to a dose-proportional accumulation in the body for 36 weeks, which was confirmed by an increased half-life, total exposure, mean residence time and plasma peak. Our data also suggests that CBD plasma levels may have less daily variation if administered twice daily.

## 1. Introduction

Hemp (*Cannabis sativa*) originated from Central Asia and has a rich history of therapeutic use by humans that dates back thousands of years B.C. (1). Although hemp has known medicinal properties, regulations in the US prohibited its use with the Marihuana Tax Act of 1937, and it has only recently become available to the public with the Farm Bill of 2018. This regulatory change increased consumer and research interest in cannabidiol (CBD), which is among the most relevant phytocannabinoids (PC) in hemp. Specifically, CBD for canine patients in Veterinary Medicine is known for being well tolerated (2–5) and for having medicinal properties in alleviating symptoms of inflammatory (6–9) and neurological (10, 11) conditions. For instance, CBD was reported to reduce pruritus in canines with atopic dermatitis (8), to improve osteoarthritis symptoms (7), and to reduce seizure frequency in addition to antiepileptic drugs in dogs with drug-resistant idiopathic epilepsy (11, 12).

When any exogenous substance of interest is being introduced to the market, it is essential to understand its pharmacological properties in order to describe its functionality in the target species. Pharmacokinetics (PK) is an effective way to communicate functionality in terms of the spatial and temporal distribution of exogenous substances in a biological system (13). Exogenous substances introduced to biological systems undergo complex kinetic changes that comprise transportation, biochemical modification, and elimination (13). Although pharmacokinetic models may be calculated based on other previously published observed models, such as comparing dog to human and vice versa, gastrointestinal anatomy and physiology differences vastly alter absorption, distribution, metabolism, and elimination in each species (14), which impact the PK of any potentially studied exogenous substance. Therefore, PK studies on the target species are necessary. During a PK, the substance is introduced to the body and timed samples are collected for subsequent measurement of the target molecule. These timed measurements allow researchers to plot a PK curve, and both observe and calculate important parameters with mathematical models.

The majority of CBD PKs in dogs have been conducted with various doses, using different oral supplementation forms or carrier oils, to subjects naïve to the drug (3, 7, 15–18). Only one study, to our knowledge, conducted CBD PK in canines at the study baseline as well as after 28 days of daily CBD dosing, and they found indication of CBD accumulation (5).

Chronic health conditions like epilepsy (11), dermatitis (8) or osteoarthritis (7) require continuous CBD dosing, making it imperative to understand how PK is affected long-term. Therefore, the goal of the present study was to measure and calculate the PK on naïve dogs to CBD, as well as sequential PKs at 18 and 36 weeks of daily CBD administration. A secondary goal was to measure CBD monthly (every 4 weeks) at trough and peak to capture the fluctuation in CBD plasma levels of dogs dosed once daily.

## 2. Materials and methods

### 2.1. Animals and study design

Eighteen (nine neutered male, nine spayed female, all Beagle breed) adult healthy dogs, average age 2.3 years ±0.14 (range 2.1 to 2.6 years old), with body weight (BW) 9.5 kg ± 1.80 (range 7.1 to 12.8 kg) at study start were randomly assigned (n = 6) to one of three treatment groups; 5 mg/kg BW CBD,10 mg/kg BW CBD and 0 mg/kg BW CBD (vehicle only). Groups were balanced by sex and body weight, and dogs belonging to the same treatment were housed in pairs or trios when necessary. The study was approved by the Institutional Animal Care and Use Committee (IACUC) at Colorado State University (protocol number 2121).

All dogs were fed controlled amounts of adult dry dog food (Hill’s Science Diet Adult Chicken & Barley Recipe; Hill’s Pet Nutrition) once daily between 07:00 and 09:00, and dosed once daily with their respective treatment oils within 30 min of feeding. Fresh water was provided *ad libitum*. Dogs were weighed every 2 weeks and both their food offered and CBD doses were adjusted accordingly. All study personnel were masked except for one of the PIs. More detailed information about housing and enrichment has been previously described (19).

### 2.2. Treatments

Two broad spectrum industrial hemp extracts were formulated to deliver 5 and 10 mg/kg CBD once daily to study dogs in similar volumes. Oils contained 5.1 and 10.0% CBD in a medium-chain triglyceride (MCT) vehicle oil, respectively, where 95% of the cannabinoid profile was CBD. There was a negative control group that received the MCT oil without hemp. Cannabidiol concentrations were measured using high performance liquid chromatography (HPLC) with UV absorption and diode array detector (DAD) at an external laboratory [SC Labs, Denver CO, United States]. The CBD determination was conducted at the beginning, middle and end of the experiment, as previously described (19). There were non-detectable levels of delta-8 and delta-9 THC in both oils.

### 2.3. Plasma CBD determinations

Plasma samples were processed and quantified at the Flint Animal Cancer Center, Drug Discovery and Development Shared Resources Core facility at Colorado State University (Fort Collins, CO, United States). Plasma extracts were prepared by liquid–liquid extraction using D3-CBD (Cerilliant Corporation, Sigma Aldrich, Round Rock, TX, United States) as the internal standard (IS) at a final concentration of 200 ng/mL. A standard curve was created in acetonitrile ranging from 0.98 ng/mL to 2000 ng/mL by spiking 100 ul blank canine plasma with 10 μL of 10x Standard at each concentration along with 10 uL of 10X D3-CBD as internal standard. One hundred microliters of sample plasma was spiked with 10 μL of 10X IS and 10 μl of acetonitrile then all samples were mixed with 500 μL ethyl acetate on a shaker for 10 min at room temperature. Samples were centrifuged for 10 min at 14,000x g to separate organic and aqueous phases, then 400 μL of organic phase from each extract was transferred to a fresh tube and evaporated to dryness in a SpeedVac on high heat for 30–40 min. The remaining pellet was reconstituted with 100 μL acetonitrile before transferring to vials with glass inserts. The CBD in processed plasma extracts was isolated by injecting 30 microliters using a LEAP autosampler onto a Waters Sunfire C18, 5 μm, 4.6 × 50 mm column, and quantified by mass spectrometry (Sciex 3,200 Q-TRAP triple quadrupole MS; Applied Biosystems, Inc., Foster City, CA, United States). The column oven was set to 30°C, flow rate set to 1,000 μL/min (LC-20 AD HPLC system, Shimadzu Corporation, Kyoto, Japan), and total run time was 7 min. The mobile phase was composed of acetonitrile with 0.1% formic acid and HPLC-grade water with 0.1% formic acid at the proportions 75:25 for 1.5 min, 99:1 for 3.5 min, and 75:25 for 2 min. Data acquisition was performed using Sciex Analyst software v1.7.1. Quantitation analysis of CBD was performed using a linear fit to calibration with a weighted least square (1/x2) regression using 12 standards.

As described in a recent CBD tolerability study (19), 0 h and 2 h post-prandial plasma collections occurred every 4 weeks for 36 weeks. Cannabidiol trough (0 h) and peak (2 h) plasma concentrations were measured. The 0 h represented time of fast and nearly 24 h after last dose, while the 2 h after feeding and dosing was an assumption that CBD peak concentrations would be captured based on previous research (7, 16, 18).

### 2.4. Pharmacokinetics

Pharmacokinetics were conducted at 3 instances during the study: at the beginning, when dogs were naïve to CBD (day 0), as well as at 18 and 36 weeks of chronic daily supplementation. During each PK, a catheter was first placed on one of the front limbs of each dog. Four mL blood were collected from the cephalic vein for timepoint 0 h, and 2 mL was transferred to a green-top tube (BD Vacutainer® sodium heparin 33 IU; BD Company, Franklin Lakes, NJ, United States) for CBD plasma determination (2 mL was stored as serum for metabolomics determination, not presented here). Dogs were fed in groups of 3 to stagger blood collections. Immediately after 10 min, food bowls were removed and dogs were dosed with their respective oils. The exact time of oil dosed was recorded, and 2 mL of blood was collected for CBD determinations at 2, 4, 8, 12, 24, and 48 h of the initial dosing time. Green-top tubes with blood were kept in ice for around 1 h until processed. Tubes were centrifuged (Avanti J-15R centrifuge; Beckman Coulter Company, Pasadena, CA, United States) at 4°C for 10 min at 2,000 G to separate plasma, which was immediately frozen at −80°C.

A non-compartmental modeling approach was used to measure PK parameters (13) and were calculated using a software (Phoenix WinNonlin™; Certara, Princeton, NJ, United States). These included time to reach maximum CBD peak (C_max_), time to reach maximum CBD peak normalized by dose (C_max_D), time at which CBD reached its peak (T_max_), half-life or the time it took for plasma CBD concentration to be reduced to half of its peak (T_1/2_), area under the curve from 0 to 48 h or total CBD exposure within this time frame (AUC_0-48h_), area under the curve from 0 to 48 h normalized by dose (AUCD_0-48h_), volume of distribution (Vz/F), clearance (Cl/F) and mean residence time (MRT). Because an intravascular arm was not included in this study, the bioavailability parameter “F” could not be calculated and is currently undetermined.

### 2.5. Statistical analysis

Data were analyzed as repeated measures over time using the generalized linear mixed model (GLIMMIX) procedure from statistical analysis software (SAS Institute v 9.4, Cary, NC). Specifically, changes in PK parameters were analyzed over 3 timepoints (0, 18, and 36 weeks), while changes in CBD plasma concentration (trough and peak) were analyzed every 4 weeks. Fixed effects were timepoint (time), treatment and their interaction. The subject was defined as dog nested within treatment, and covariance structure was defined as unstructured (UN) for plasma CBD and compound symmetry (CS) for PK parameters based on the Bayesian information criterion (BIC). When data did not meet model assumptions assessed by studentized residuals plot, natural logarithm transformation was performed, and data were back transformed to the original scale for reporting. Pairwise treatment comparisons were adjusted with Tukey–Kramer post-hoc test to protect against type I error. Significance was noted at an *α* = 0.05.

## 3. Results

### 3.1. Pharmacokinetics

**Figure 1:**
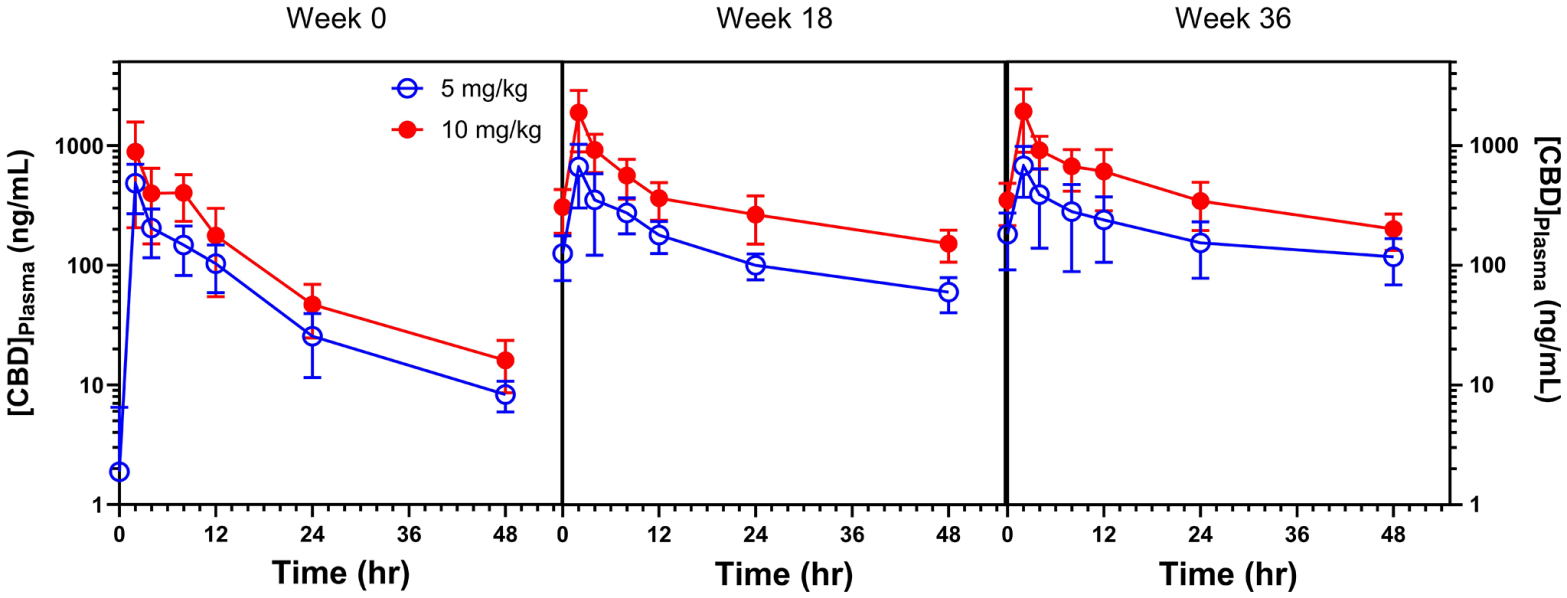
Pharmacokinetic curves (mean ± standard error) of dogs administered 5 and 10 mg/kg CBD at week 0 (baseline, naïve to CBD), and at weeks 18 and 36 of chronic daily CBD supplementation.

The 48 h PK parameters were compared both between treatments (5 and 10 mg/kg CBD) and across time (weeks 0, 18, and 36). The placebo group had non-detectable to negligible CBD levels throughout the entire study, as expected, and thus was not reported. A visual representation of the 3 PKs was plotted (Figure 1). There was clear evidence for a cumulative effect of CBD, as well as dose-proportional magnitude changes. Cannabidiol C_max_ was approximately twice as high in 10 versus 5 mg/kg dose treatment group when administered to naïve dogs, and nearly 3-fold greater in 10 vs. 5 mg/kg at weeks 18 and 36 [Table 1; *P* (Treatment) = 0.003 and *P* (Time) = 0.005]. When C_max_ was normalized by dose (C_max_D), the treatment effect was lost but plasma CBD concentrations increased over each time of PK collection [*P* (Time) = 0.005]. Likewise, AUC_0-48h_ at week 0 was approximately twice as high in dogs given 10 mg/kg vs. 5 mg/kg, and this difference increased over time [*P* (Treatment) = 0.001 and *P* (Time) < 0.0001]. The AUC_0-48h_ normalized by dose (AUC_0-48h_D), similar to C_max_D, showed a clear increase in total CBD exposure at both weeks 18 and 36 [*P* (Time) < 0.0001]. The time to reach C_max_ was 2 h for most dogs, with a few exceptions where the CBD maximum observed level was closest to 8 or 12 h. Half-life (T_1/2_) was similar between treatments [*P* (Treatment) = 0.459] and increased with chronic dosing from week 0 to 18, plateauing from week 18 to 36 [*P* (Time) < 0.0001]. One dog in group 5 mg/kg had a calculated T_1/2_ of 316 h at the third PK (week 36), so it was considered an outlier and removed from statistical analysis.

**Table 1:** Non-compartmental pharmacokinetics parameters means [95% CI] of beagles continuously dosed with 5 and 10 mg/kg CBD (*n* = 6) once daily for 36 weeks.

	Week 0		Week 18		Week 36		*P*	*P*
Treatment	5 mg/kg	10 mg/kg	5 mg/kg	10 mg/kg	5 mg/kg	10 mg/kg	(Treatment)	(Time)
C_max_, ng/mL	441^bx^ [284, 686]	880^ax^ [566, 1,369]	585^by^ [376, 909]	1,709^ay^ [1,099, 2,657]	616^by^ [396, 957]	1,746^ay^ [1,123, 2,714]	0.003	0.005
C_max_D, kg*ng/mL/mg	88.2^x^ [56.8, 137.2]	88.0^x^ [56.6, 136.9]	116.9^y^ [75.2, 181.8]	170.9^y^ [109.9, 265.7]	123.1^z^ [79.2, 191.4]	174.6^z^ [112.3, 271.4]	0.342	0.005
AUC_0-48h_, h*ng/mL	3,525^bx^ [2,650, 4,690]	6,930^ax^ [5,209, 9,220]	7,205^by^ [5,415, 9,586]	18,033^ay^ [13,554, 23,992]	9,346^bz^ [7,025, 12,434]	22,138^az^ [16,640, 29,454]	0.001	<0.0001
AUCD_0-48h_, hr.*kg*ng/ml/mg	705^x^ [530, 938]	693^x^ [521, 922]	1,441^y^ [1,083, 1917]	1,803^y^ [1,355, 2,399]	1,869^z^ [1,405, 2,487]	2,214^z^ [1,664, 2,945]	0.467	<0.0001
^1^T_max_, h	3.67 ± 4.082 (2–12)	3 ± 2.45 (2–8)	2 ± 0.0 (2–2)	2 ± 0.0 (2–2)	2 ± 0.0 (2–2)	3 ± 2.45 (2–8)	–	–
^2^T_1/2_, h	8.8^y^ [6.2, 12.5]	12.6^y^ [8.8, 17.9]	24.6^z^ [17.3, 35.0]	28.3^z^ [19.9, 40.3]	30.6^z^ [20.7, 45.3]	26.9^z^ [18.9, 38.2]	0.459	<0.0001
Vz/F, L/kg	18.0 [10.5, 30.9]	26.2 [15.2, 44.9]	24.6 [14.3, 42.2]	22.7 [13.2, 38.9]	35.1 [20.5, 60.3]	17.5 [10.2, 30.1]	0.597	0.856
CL, L/h/kg	1.418^x^ [1.066, 1.887]	1.444^x^ [1.085, 1.921]	0.694^y^ [0.522, 0.923]	0.555^y^ [0.417, 0.738]	0.535^z^ [0.402, 0.712]	0.452^z^ [0.340, 0.601]	0.468	<0.0001
MRT_0-48h_	9.93^x^ [7.82, 12.04]	11.11^x^ [9.00, 13.22]	14.91^y^ [12.80, 17.02]	14.63^y^ [12.52, 16.74]	17.82^z^ [15.71, 19.93]	15.50^z^ [13.39, 17.61]	0.697	<0.0001

Pharmacokinetics was conducted at weeks 0, 18, and 36, and effects of treatment and time are shown below.

^a,b^Different superscripts denounce significance between treatments within each parameter in a row.

^x–z^Different superscripts denounce significance between timepoints within each parameter in a row.

^1^Mean ± Standard deviation (range) was reported for T_max_ due to non-parametric data.

^2^One outlier in the 5 mg/kg CBD treatment at week 36 was removed from the table.

Volume of distribution (Vz/F) was similar between treatments and did not change over time, ranging from 17.5 to 35.1 L/kg (Table 1). Clearance (CL) decreased over time [*P* (Time) < 0.0001], whereas MRT increased at both weeks 18 and 36 [*P* (Time) < 0.0001].

### 3.2. Monthly plasma CBD

**Figure 2:**
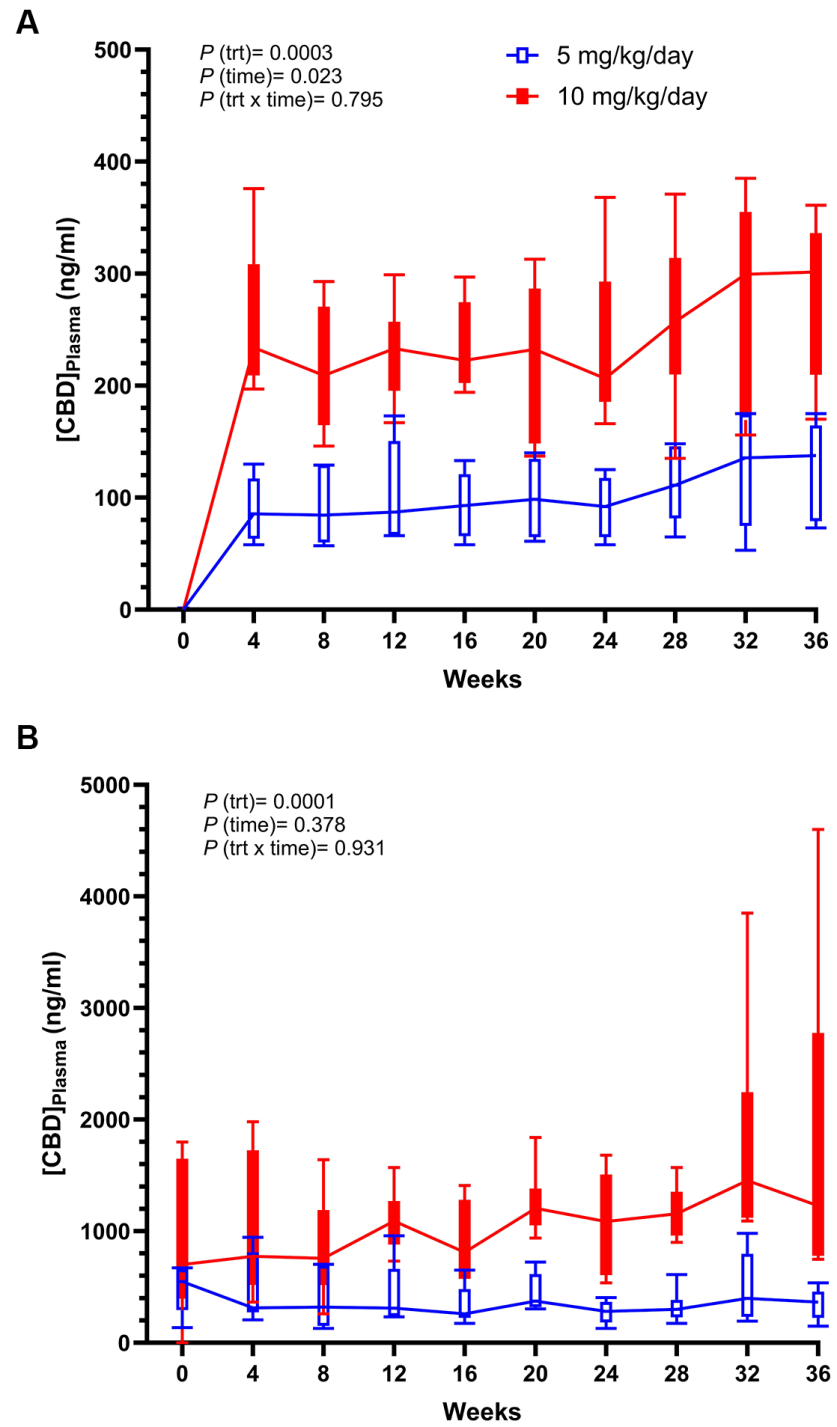
Box plots of trough (0 h) **(A)** and peak (2 h) **(B)** plasma CBD measured over time of dogs supplemented daily with 10 mg/kg CBD (solid red) and 5 mg/kg CBD (unfilled blue). *p*-values reported include treatment (trt), time, and treatment by time (trt x time).

Plasma CBD was determined at its hypothesized trough level (at fast, nearly 24 h after its last dose, Figure 2A), and close to its peak at 2 h after feeding and dosing, Once every 4 weeks for 36 weeks (Figure 2B). The concentration of CBD present in plasma averaged from 104 to 390 ng/mL in dogs supplemented with 5 mg/kg CBD, and from 246 to 1,216 ng/mL in dogs supplemented with 10 mg/kg CBD. There were markedly treatment differences at both trough (*p* = 0.0003) and peak (*p* = 0.0001) collections, and only CBD trough levels had a significant time effect (Figure 2A), although pairwise comparisons between months did not show a difference after Tukey adjustment. The monthly variation in plasma CBD at 2 h collection among dogs receiving the same treatment was greater than that at fast, and dogs receiving 10 mg/kg/d CBD had the greatest variation at weeks 32 and 36 (Figure 2B).

## 4. Discussion

The primary goal of the present work was to quantify PK changes over time in orally supplemented dogs with CBD at 5 and 10 mg/kg using an MCT oil vehicle. There was a placebo group not presented in this work that served as negative control for a previous CBD tolerability study (19). The target dose 5 mg/kg was chosen based on what has been previously studied in dogs with idiopathic epilepsy (11), osteoarthritis (7, 9), and anxiety in humans (20). The 10 mg/kg dose was chosen as in previous studies also showed tolerability and safety (7, 15), with differences only found with an increased C_max_ compared with lower CBD dosage (7). There have been some single-dose PK studies in dogs (3, 7, 15–18) that were conducted over 24 h using different CBD oral formulations and doses, whereas the present work performed multiple 48 h-PKs during long-term administration. To the authors’ knowledge, there are only two studies to date that investigated the effects of repeated CBD doses on PK parameters of dogs (5, 21) which had some common findings to the present work that will be discussed in more detail below.

When administered to naïve dogs, broad spectrum hemp extracts containing mainly CBD present differences in PK parameters across studies that can be attributed to factors like vehicle choice (15, 18), fed versus fasted state of dogs (17, 22, 23) and PK length. For instance, when comparing two oils and a soft chew with similar concentrations of CBD, (18) found that the solid form had a delayed and higher absorption peak relative to the oils. Similarly, three different CBD delivery formats (oil, capsule and dermal cream) had a much greater impact on PK, affecting most parameters (15). Since each hemp formula and format are metabolized differently, direct comparisons among studies must be made with caution. When supplementing the same doses as the current study, but using a herbal extract containing 1:20 THC:CBD (16), CBD C_max_ were reported to be twice as high.

There have been a few studies that reported a CBD dose-dependent C_max_ and AUC (7, 16), similar to what was observed in the present study. This dose effect does not seem to be linear, as CBD administered at 20 mg/kg in dogs (17) and over 3,000 mg in humans (24) did not reflect proportional increases in C_max_ and AUC relative to smaller doses. Dogs in the present work seemed to fall within the linear window of a dose magnitude effect on C_max_ and AUC at 5 and 10 mg/kg/d CBD. When normalizing both C_max_ and AUC_0-48h_ by dose, our work clearly showed a cumulative effect of CBD over time, which was also reported in dogs (5) and rats (25) after 28 days of daily CBD supplementation. The latter also found significant PK differences between males and females (25), which were not evidenced in the present study because dogs in each treatment were balanced by sex, age and weight. However, we were able to compare sex differences herein and found that Cl/F was higher in females (*p* = 0.003), and Cmax_D had a tendency (*p* = 0.087) to be higher in males.

The novelty about the present work is that values of AUCD_0-48h_, AUC_0-48h_, and C_max_D increased from week 0 to 18, as well as from week 18 to 36, indicating continuous accumulation over a long period of time. These results could be expected because cannabinoids are highly lipophilic (25, 26), and CBD was observed to accumulate 10–100 fold greater in adipose tissue than in hepatic or muscular tissues of rats (25). Thus, cannabidiol accumulation in adipose tissue would also be expected to occur in dogs. Long-term increases in C_max_ and AUC_0-48h_ could be a consequence of CBD being mobilized from adipose tissue, but this was not measured. Trough and peak CBD plasma levels did not increase over time to corroborate PK findings, what emphasizes the importance in conducting the 48-h PKs. Like CBD measured at trough and peak timepoints, the average plasma CBD measured at the same timepoints during the 3 PKs in Figure 1 were at similar levels; however, the slopes between weeks 0 and 18 had a drastic change, and the negative slope had a further decrease at week 36 PK. These changes in slopes led to the increases in AUCD_0-48h_ and AUC_0-48h_.

Cannabidiol’s T_max_, or time to reach its plasma peak, has been relatively consistent among studies (3, 5, 7, 16, 18) at 1 to 4 h, and is not influenced by dose or chronic administration according to both the present work and (18). Conversely, half-life has been reported to widely vary among dogs, and to range from nearly 1 to 24 h in single-dose PK studies (5, 7, 15, 16, 18). Elimination T_1/2_ is a dependent variable directly related to volume of distribution and inversely related to clearance, which are both independent variables (27). Although T_1/2_ does not have much value in predicting drug elimination with a single dose, it is valuable in predicting the rate of drug accumulation and elimination after consecutive doses (27). In the present study, half-life almost tripled after 18 weeks, and remained similar at week 36, strengthening the argument that CBD accumulates in dogs over time. This also corroborates with previous findings in regard to a half-life increase after 28d of CBD administration twice daily at various doses (5).

Volume of distribution via extravascular (Vz/F) is calculated as the collective amount of a compound present in the body that was absorbed over the PK. It can be defined as the volume of plasma that would be necessary to account for the total amount of drug in the patient’s body, if that exogenous substance were present throughout the body at the same concentration as found in the plasma. A high Vz/F indicates that the substance or drug is extensively distributed in other tissues rather than present in the blood (27). Volume of distribution of CBD found in dogs in the present work was comparable to what has been reported in humans (24) and horses (28). In contrast, volume of CBD distribution in cats was reported to be higher (29); although there is limited research in cats, this may indicate that cats and dog Vz/F cannot be compared. Clearance (CL) refers to the hypothetical volume of plasma from which a drug is completely removed per hour (27), and in this study, it decreased over 36 weeks indicating a lower rate of CBD elimination with chronic administration. The CL rate was also similar in horses (28) at the week 0 timepoint in this study. Cannabidiol accumulation and rate of elimination may be attributed to its lipophilic nature, as well as to anatomical differences of mammals. For instance, the adipose distribution of fascia in both dogs and humans have a superficial adipose tissue, whereas the horse fascia lacks this adipose layer (30). Thus, if CBD was administered to horses long-term, we could expect consecutive CL measurements to differ from the dog due to a greater accumulation in the fascia superficial adipose tissue of canines. This theory would need scientific evidence to be validated. Finally, MRT refers to the average time CBD spends in the body before being eliminated (13), and in this study MRT also reflected CBD accumulation over time. Although both CL and MRT indicated CBD storage mostly in adipose tissue, Vz/F was unaffected by time. This lack of significance could have happened because of the high intraspecies variation, as well as due to Vz/F calculation that does not account for the ratio of CBD distribution among body tissues.

A secondary goal of the present study was to determine plasma CBD concentrations over 36 weeks. Plasma CBD presented monthly variations when measured both at trough and peak (pre-prandially and 2 h pos-feeding and dosing). After the first month (week 4) of chronic administration, CBD had already reached high levels. It might be possible that plasma CBD had a weekly incline during the first month, similar to what was (2) reported, but this was not captured here. High intragroup variation was found in the current study and also corroborates previous plasma CBD research (2). In their work CBD peak levels were not measured, so they could only assume what it was before these were measured in the current study. After nearly 24 h of dosing, plasma CBD levels dropped 3.8–4.9 times that of its supposed peak. It has been suggested that CBD plasma levels correlates with a reduction in seizure activity in dogs (11), so it might be necessary to administer CBD twice daily to maintain consistent therapeutic CBD plasma levels. Twice daily chronic dosing might also lower the impact on liver enzymes such as ALP, which has been vastly reported to increase in dogs taking CBD (2, 4, 5, 7, 17, 31) but this still needs scientific evidence. Dosing recommendations including frequency for chronic use should be further investigated in regard to how it may influence clinical outcomes of dogs.

Some study strengths included sample size, PK duration, and repeated PKs over a long-time interval. Although sample size may be deemed a small representation (*n* = 6), the repeated measures for each treatment at 3 timepoints allowed it to be sufficient to detect both treatment and time differences, with a power of 93% for treatment and 86% for time based on AUC_0-48h_, determined by the GLMPOWER procedure from SAS (v 9.4).

A study limitation was that intravenous CBD AUC was not determined, and that would be necessary to calculate absolute bioavailability. A single-PK study with 8 horses dosed at 10 mg/kg CBD found oral bioavailability to be low (14%) for CBD formulated with sesame oil as the vehicle (28). Likewise, a study in humans found that single doses of 3 forms of oral CBD had <7% bioavailability, and it was lower in fasted than fed states (24). Doran et al. (17) reported that both C_max_ and AUC were greater in fed vs. fasted dogs, which indicates that dosing dogs at a fed state in the current study likely contributed to a higher CBD absorption and bioavailability. Although bioavailability of any oral CBD suspension has not yet been determined for canines to the authors’ knowledge, it would be expected to be low if we extrapolate what has been found in other monogastric animals (24, 28). Future studies should focus on determining CBD absolute bioavailability, as well as understand the effect of CBD that bypasses small intestine digestion on the colonic microbiome of dogs.

## Data availability statement

The raw data supporting the conclusions of this article will be made available by the authors, without undue reservation.

## Ethics statement

The animal study was approved by Institutional Animal Care and Use Committee (IACUC) at Colorado State University (protocol number 2121). The study was conducted in accordance with the local legislation and institutional requirements.

## Author contributions

IC: Conceptualization, Data curation, Formal analysis, Investigation, Methodology, Project administration, Writing – original draft. DG: Data curation, Methodology, Software, Validation, Visualization, Writing – review & editing. KB: Data curation, Methodology, Validation, Writing – review & editing. KW: Conceptualization, Funding acquisition, Methodology, Resources, Supervision, Writing – review & editing. SM: Conceptualization, Funding acquisition, Investigation, Methodology, Project administration, Resources, Supervision, Validation, Writing – review & editing.
